# The prognostic value of left ventricular systolic function measured by tissue Doppler imaging in septic shock

**DOI:** 10.1186/cc11328

**Published:** 2012-05-03

**Authors:** Li Weng, Yong-tai Liu, Bin Du, Jian-fang Zhou, Xiao-xiao Guo, Jin-min Peng, Xiao-yun Hu, Shu-yang Zhang, Quan Fang, Wen-ling Zhu

**Affiliations:** 1 Department of Cardiology, Peking Union Medical College Hospital, Peking Union Medical College and Chinese Academy of Medical Sciences, 1 Shuaifuyuan, Dongcheng district, Beijing, 100730, China; 2 Medical ICU, Peking Union Medical College Hospital, Peking Union Medical College and Chinese Academy of Medical Sciences, 1 Shuaifuyuan, Dongcheng District, Beijing, 100730, China

## Abstract

**Introduction:**

Left ventricular (LV) dysfunction is common in septic shock. Its association with the clinical outcome is still controversial. Tissue Doppler imaging (TDI) is a useful tool to quantify LV function; however, little knowledge is available about the prognostic value of these TDI variables in septic shock. Therefore, we performed this prospective study to determine the role of TDI variables in septic shock.

**Methods:**

Patients with septic shock in a medical intensive care unit were studied with transthoracic echocardiography with TDI within 24 hours after the onset of septic shock. Baseline clinical, laboratory, and echocardiographic variables were prospectively collected. Independent predictors of 90-day mortality were analyzed with the Cox regression model.

**Results:**

During a 20-month period, 61 patients were enrolled in the study. The 90-day mortality rate was 39%; the mean APACHE IV score was 84 (68 to 97). Compared with survivors, nonsurvivors exhibited significantly higher peak systolic velocity measured at the mitral annulus (Sa) (11.0 (9.1 to 12.5) versus 7.8 (5.5 to 9.0) cm/sec; *P *< 0.0001), lower PaO_2_/FiO_2 _(123 (83 to 187) versus 186 (142 to 269) mm Hg; *P *= 0.002], higher heart rate (120 (90 to 140) versus 103 (90 to 114) beats/min; *P *= 0.004], and ahigher dose of norepinephrine (0.6 (0.2 to 1.0) versus 0.3 (0.2 to 0.5) μg/kg/min; *P *= 0.007]. In the multivariate analysis, Sa > 9 cm/sec (hazard ratio (HR), 5.559; 95% confidence interval (CI), 2.160 to 14.305; *P *< 0.0001), dose of norepinephrine (HR, 1.964; 95% CI, 1.338 to 2.883; *P *= 0.001), and PaO_2_/FiO_2 _(HR, 0.992; 95% CI, 0.984 to 0.999; *P *= 0.031) remain independent predictors of 90-day mortality in septic-shock patients.

**Conclusions:**

Our study demonstrated that LV systolic function as determined by TDI, in particular, Sa, might be associated with mortality in patients with septic shock.

## Introduction

Although left ventricular (LV) depression in sepsis was first reported decades ago [1], it has not been well recognized until the recent widespread use of echocardiography in the intensive care unit (ICU) [2]. A variety of echocardiographic parameters have been developed to assess LV function [3]. Among these parameters, ejection fraction (EF) is most commonly used to evaluate LV systolic function, although studies exploring its association with clinical outcome have demonstrated conflicting results in high-risk patients, especially in patients with septic shock [2].

Tissue Doppler imaging (TDI) has been shown to be useful for quantifying global systolic and diastolic LV function [4-6]. The peak systolic velocity measured at the mitral annulus (Sa) reflects the long-axis systolic motion of the ventricle, whereas the early diastolic velocity of the mitral annulus (Ea) reflects the rate of myocardial relaxation. Both Sa and Ea have been demonstrated as useful tools to predict prognosis in a variety of cardiovascular diseases [7]. However, the prognostic value of the TDI variables in septic shock requires further clarification.

Therefore, we performed a prospective, observational study to evaluate the prognostic significance of TDI variables in septic shock.

## Materials and methods

### Patients

The study was performed in a nine-bed medical ICU of a university teaching hospital. Between January 2010 and August 2011, all patients admitted for septic shock that developed within 24 hours before ICU admission were prospectively screened for eligibility. Sepsis, severe sepsis, and septic shock were defined according to consensus definition [8] (see Additional file 1 Figure S2), and the differentiation between infectious and noninfectious etiologies was made at the discretion of the ICU consultant. In patients with multiple episodes of septic shock, only the first episode was included in this study.

Exclusion criteria included age younger than 18 years; pregnancy; presence of moderate to severe valvular heart disease; patients or their relatives declined participation; suboptimal echocardiograms; postthoracic operation; documented myocardial infarction at any point in the medical history; and a decision of withdraw or withhold life-sustaining therapy.

Baseline clinical variables during the first 24 hours after admission (day 1) were collected prospectively, including age, gender, comorbidities, hemodynamic parameters, vasopressor or inotropic dose, Acute Physiology and Chronic Health Evaluation (APACHE) IV score [9], and Sequential Organ Failure Assessment (SOFA) score [10].

### Echocardiographic examination

Two-dimensional conventional Doppler echocardiography and TDI studies were performed with commercially available equipment (Vivid I; GE Vingmed Ultrasound, Tirat Hacarmel, Israel). All studies were performed and reviewed by cardiologists with advanced training in echocardiography.

The transthoracic echocardiographic examination was performed within 24 hours after the onset of septic shock at the first day of ICU stay (day 1). LV end-diastolic volume (LVEDV), LV end-systolic volume (LVESV), and LV ejection fraction (LVEF) were assessed by using the modified biplane Simpson equation in the apical four- and two-chamber views, according to the American Society of Echocardiography Guidelines [11]. Mitral inflow was assessed with pulsed-wave Doppler echocardiography from the apical four-chamber view. The Doppler beam was aligned parallel to the direction of flow, and a 1- to 2-mm sample volume was placed between the tips of mitral leaflets during diastole [12]. From the mitral inflow profile, the E- and A-wave velocity and the E/A velocity ratio were measured. At least three consecutive beats were measured, and the average value was taken. In patients with tachycardia, the fused EA wave was considered an E wave to calculate the E/Ea.

TDI was performed at the apical four-chamber view for the long-axis motion of the heart [13,14]. Two-dimensional echocardiography with color-TDI imaging was performed. The imaging angle was adjusted to ensure a parallel alignment of the sampling window with the myocardial segment of interest. Gain settings, filters, pulse repetitive frequency, sector size, and depth were adjusted to optimize color saturation. The frame rate was adjusted to > 100. At least three consecutive beats were stored, and the images were digitized and analyzed off-line by EchoPac software (EchoPac 6.3.6; Vingmed-General Electric, Horten, Norway). Pulse-Doppler sample volume was placed at the septal and lateral MV annulus to obtain the average value of systolic (Sa) and early diastolic velocity (Ea) (see Additional file 1 Figure S2).

The intraobserver and interobserver variability in the measurements of Sa were 1.8% and 4.2%, respectively.

### Follow-up

Follow-up was performed for 90 days after the onset of septic shock. The primary end point was 90-day all-cause mortality, defined as death within 90 days after onset of septic shock. Death was identified from hospital records or telephone interviews with relatives.

The Institutional Review Board of Peking Union Medical College Hospital approved this study protocol. Written informed consent was obtained from either the patients or their authorized relatives.

### Statistical analysis

Deviations from a gaussian distribution were tested by the Kolmogorov-Smirnov test. Continuous variables were presented as median (25th to 75th percentiles). Categoric variables were expressed as percentages of the group from which they were derived. Continuous variables were compared with the use of the Student *t *test or Mann-Whitney test. Categoric variables were compared with the χ^2 ^test or Fisher Exact test. Linear regression was used to investigate the correlation between EF and Sa. A receiver-operating characteristic (ROC) curve analysis was performed to determine the cutoff value of Sa for the prediction of 90-day mortality. The optimal cut-off value was defined as the point at which the value of "sensitivity + specificity - 1" was maximum (Youden index [15,16]).

A survival curve was performed by using the Kaplan-Meier method, and mortality rates were compared according to the cut-off value of Sa by using the log-rank test.

Cox proportional hazards regression model was used to estimate the risk of death by multivariate analysis (backward stepwise selection method with probability for the removal of 0.10) for the whole population. The multivariate analysis selection criterion from the univariate analysis was *P *value < 0.05 and absence of collinearity. Collinearity was defined as variance inflation factor (VIF) > 10 by using linear regression analysis.

All analysis was performed by using software (SPSS for Windows 11.5; SPSS, Chicago, IL, USA). Statistical significance was considered at *P *< 0.05.

## Results

### Baseline characteristics

During the study period, from January 2010 to August 2011, 132 patients with septic shock were eligible for assessment. Seventy-one were excluded in the final analysis, including consent refusal (*n *= 24), severe regurgitation (*n *= 10), postthoracic operation (*n *= 5), withheld or withdrawn therapy (*n *= 14), and suboptimal echocardiograms (*n *= 18). As a result, 61 patients were analyzed. Patients excluded had higher proportion of coronary heart disease. No significant difference was noted in other baseline characteristics between patients excluded and included (see Additional file 1 Table S1).

The 33 (54%) men had a median age of 68 (52 to 77) years, and an APACHE IV score of 84 (68 to 97). Twenty-two patients died during ICU stay, and 24 patients died at 90 days after the onset of septic shock, with a 90-day all-cause mortality of 39%. Five patients died within 48 hours of ICU admission. A total of 36 (59%) patients had documented comorbidities, including coronary heart disease (13%), hypertension (46%), and diabetes (28%). For all patients, no episode of active ischemia was documented in the last 3 months before inclusion. The most common infection was pneumonia (56%). All patients required vasoactive medications to maintain blood pressure, and all were mechanically ventilated because of acute lung injury. Table 1 compared baseline clinical variables on ICU admission (day 1) between survivors and nonsurvivors. Nonsurvivors had significantly lower PaO_2_/FiO_2 _(123 (83 to 187) versus 186 (142 to 269) mm Hg; *P *= 0.002) than did survivors.

**Table 1:** Baseline characteristics and comparison between survivors and nonsurvivors at the onset of septic shock (day 1)

	Survivors (*n *= 37)	Nonsurvivors (*n *= 24)	*P *value
Characteristics			
Age, years	68 (49-76)	74 (61-82)	0.167
Male, *n *(%)	18 (49)	15 (63)	0.289
BMI, kg/m^2^	23 (21-25)	23 (20-26)	0.732
APACHE IV score	79 (66-94)	93 (69-99)	0.339
APACHE IV predicted mortality, %	28 (17-53)	48 (35-61)	0.039
SOFA score	10 (8-12)	10 (8-12)	0.222
PaO_2_/FiO_2_, mm Hg	186 (142-269)	123 (83-187)	0.002
SOFA cardiovascular score	4 (4-4)	4 (4-4)	0.911
Days on vasoactive medications	5 (3-9)	7 (4-9)	0.340
ICU LOS, days	12 (8-22)	12 (4-20)	0.515
Hospital LOS, days	29 (17-49)	17 (6-52)	0.150
Comorbidities			
Coronary heart disease, *n *(%)	6 (16)	2 (8)	0.373
Hypertension, *n *(%)	18 (49)	10 (42)	0.593
Diabetes, *n *(%)	12 (32)	5 (21)	0.324
Chronic renal failure, *n *(%)	6 (16)	2 (8)	0.373
Primary diagnosis of infection			
Pneumonia, *n *(%)	18 (49)	16 (67)	0.166
Bacteremia, *n *(%)	5 (14)	2 (8)	0.535
Peritonitis, *n *(%)	5 (13)	1 (4)	0.231
Others, *n *(%)	9 (24)	5 (21)	0.751
Laboratory data			
Lactate, m*M *	1.75 (1.30-2.88)	2.00 (1.80-3.98)	0.095
WBC, ×10^9^/L	11.36 (7.18-19.99)	12.07 (6.91-18.90)	0.623
Procalcitonin, ng/ml	2.01 (0.59-7.40)	1.50 (0.53-5.71)	0.435
cTnI, μg/L	0.17 (0.06-1.14)	0.15 (0.04-0.93)	0.952
CKMB, μg/L	1.80 (0.60-3.83)	1.65 (1.03-4.90)	0.455
NTproBNP, pg/ml	4,072.00 (2,006.50-11,885.50)	3,710.00 (1,361.50-10,618.25)	0.693

APACHE, Acute Physiology And Chronic Health Evaluation; BMI, body mass index; Hospital LOS, hospital length of stay; SOFA, Sequential Organ Failure Assessment.

Table 2 summarized hemodynamic and echocardiographic parameters on day 1. Heart rate was significantly higher in nonsurvivors (120 (90 to 140) versus 103 (90 to 114) beats/min; *P *= 0.004]. Forty-nine (80%) patients received norepinephrine infusion at a median dose of 0.5 (0.2 to 0.7) μg/kg/min. The dose of norepinephrine was significantly higher in nonsurvivors (0.6 (0.2 to 1.0) versus 0.3 (0.2 to 0.5) μg/kg/min; *P *= 0.007]. Twenty-seven (44%) patients were treated with dopamine, the median dose being 6.0 (5.0 to 10.0) μg/kg/min. During the study periods, one patient was treated with dobutamine (5 μg/kg/min), and another, with epinephrine (0.5 μg/kg/min).

**Table 2:** Baseline hemodynamic and echocardiographic data of survivors and nonsurvivors at the onset of septic shock (day 1)

	Survivors (*n *= 37)	Non-survivors (*n *= 24)	*P *value
Hemodynamic parameters			
Heart rate, beats/min	103 (90-114)	120 (90-140)	0.004
Mean arterial pressure, mm Hg	76 (74-83)	74 (70-82)	0.189
Central venous pressure, mm Hg	16 (12-17)	14 (11-19)	0.911
Dopamine, *n *(%)	17 (46)	10 (42)	
Dose, μg/kg/min	5.0 (4.0-10.0)	6.0 (4.5-10.0)	0.836
Norepinephrine, *n *(%)	30 (81)	19 (79)	
Dose, μg/kg/min	0.3 (0.2-0.5)	0.6 (0.2-1.0)	0.007
Balance on day 0, ml/24 hours	1,180 (445-2,140)	1,850 (15-3,004)	0.640
Echocardiographic data			
Systolic parameters			
LVEDV, ml	72 (54-98)	63 (56-78)	0.110
LVESV, ml	30 (20-52)	26 (19-34)	0.169
LVEF biplane, %	56 (36-65)	63 (52-66)	0.111
LVEF biplane < 50%, *n *(%)	12 (33)	4 (17)	0.234
Sa, cm/sec	7.8 (5.5-9.0)	11.0 (9.1-12.5)	< 0.0001
Sa > 9 cm/s, *n *(%)	6 (17)	18 (75)	< 0.0001
Diastolic parameters			
E/A	0.9 (0.7-1.4)	0.7 (0.6-1.2)	0.171
Ea, cm/sec	8.3 (5.8-10.0)	7.0 (6.0-11.0)	0.634
Ea < 8 cm/sec, *n *(%)	15 (42)	12 (52)	0.429
E/Ea	11.1 (8.5-14.6)	11.1 (6.6-14.1)	0.206

E, peak velocity of early diastolic transmitral flow; Ea, early diastolic velocity of the mitral annulus; E/A, the ratio of mitral valve peak E-wave and peak A-wave velocity; EDT, deceleration time of mitral E wave; LVEDV, left ventricular end-diastolic volume; LVEF, left ventricle ejection fraction; LVESV, left ventricular end-systolic volume; Sa, peak systolic velocity measured at mitral annulus.

### Echocardiographic variables

**Figure 1:**
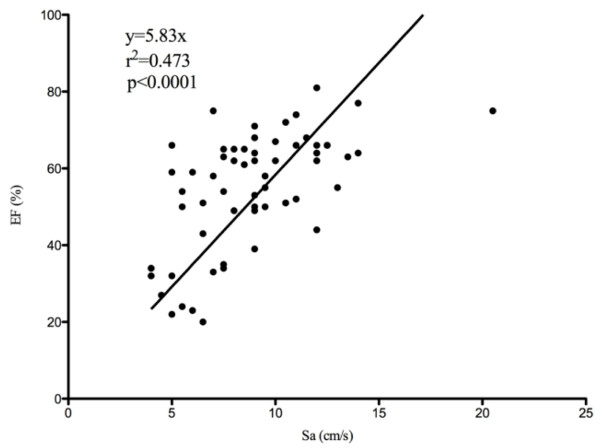
**The line regression between left ventricular ejection fraction (LVEF) and mitral annulus (Sa)**.

Five patients were in atrial fibrillation at the time of echocardiography study. Ten of 61 patients had fused E A wave, six in the group of survivors. Sa was significantly lower in the survivors group than in the nonsurvivors (7.8 (5.5 to 9.0) versus 11.0 (9.1 to 12.5) cm/sec; *P *< 0.0001), with a mean value of 9.0 (6.6 to 11.0) cm/sec for the whole cohort. Sixteen (27%) patients had an LVEF < 50%. LVEF values for survivors and nonsurvivors were 56% (36% to 65%) and 63% (52% to 66%), respectively. Other parameters, including those evaluating diastolic function, did not show any statistical difference between survivors and nonsurvivors (Table 2). A moderate correlation between LVEF and Sa was identified with linear regression (Figure 1).

**Figure 2:**
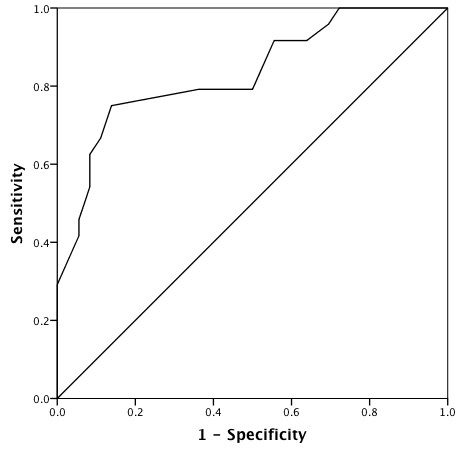
**Receiver-operating characteristic (ROC) curve for predicting 90-day mortality by using the peak systolic velocity measured at the mitral annulus (Sa)**. Area under the curve is 0.83.

**Figure 3:**
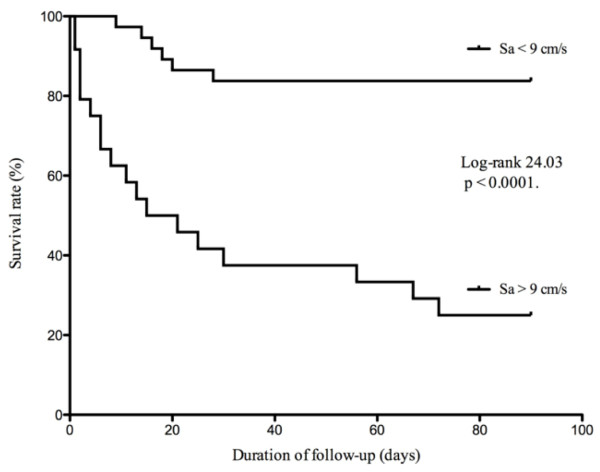
**The 90-day mortality in the study population classified according to the peak systolic velocity measured at mitral annulus (Sa) < 9 cm/sec or Sa > 9 cm/sec**.

The ability of Sa to predict 90-day mortality according to an ROC curve is shown in Figure 2, the area under the curve being 0.83. With a cut-off value of 9 cm/sec, the sensitivity and specificity of Sa to predict 90-day mortality was 75% and 86%, respectively. Patients with a higher Sa value (> 9 cm/sec) had a significantly higher mortality rate (75% versus 17%; *P *< 0.0001; log-rank = 24.03; *P *< 0.0001) (Table 2 and Figure 3).

### Predictors of 90-day mortality in septic shock patients

In the final multivariate analysis, Sa > 9 cm/sec remained the strongest independent predictor of 90-day mortality in septic shock patients (HR, 5.559; 95% CI, 2.160 to 14.305; Wald, 12.652; *P *< 0.0001). Moreover, norepinephrine dose and PaO_2_/FiO_2 _were also independent predictors, whereas heart rate did not exhibit any predictive value. Subgroup analysis showed that, in patients without a history of coronary heart disease, a Sa value of > 9 cm/sec was still an independent predictor of 90-day mortality (HR, 4.546; 95% CI, 1.749 to 11.819; Wald, 9.651; *P *= 0.002) (Table 3).

**Table 3:** Multivariate analysis for predictors of death in patients with septic shock

	Multivariate analysis		
	Hazard ratio (95% CI)	**Wald stat**.	*P *value
PaO_2_/FiO_2 _	0.992 (0.984-0.999)	4.628	0.031
Norepinephrine, μg/kg/min	1.964 (1.338-2.883)	11.898	0.001
Sa > 9 cm/sec	5.559 (2.160-14.305)	12.652	< 0.0001

## Discussion

The major finding of our study was that increased Sa is an independent predictor of 90-day mortality in patients with septic shock.

In the landmark study of Parker *et al. *[1], 10 of the 20 patients with septic shock exhibited global hypokinesia and ventricular dilation during the first 48 hours after admission. Contrary to common sense, the authors found significantly impaired LV systolic function in survivors compared with nonsurvivors. Subsequent studies [17,18] demonstrated similar reversible global hypokinesia by echocardiography. Vieillard-Baron and colleagues [17] observed global hypokinesia in 26 of 67 patients. Moreover, LVEF was compromised in survivors during the first 24 hours (49% ± 18% versus 55% ± 15%) [17]. In another study performed over a period of 5 years [18], survivors showed evidence of septic myocardial dysfunction, as suggested by compromised LVEF (43.9% ± 16.4% versus 52.0% ± 14.0%) and higher LVEDV (75.3 ± 20.1 ml/m^2 ^versus 64.9 ± 25.0 ml/m^2^; *P *< 0.05). However, similar to our study, the difference in LVEF between survivors and nonsurvivors was not significantly different.

The linear correlation between Sa and ejection in our study was similar to that of a previous study [5]. Unlike LVEF, Sa was a sensitive marker of LV systolic function in patients with cardiovascular disease, which showed that Sa could predict clinical outcome in a more sensitive manner than could the LVEF [7]. In a study of hypertrophic cardiomyopathy, TDI revealed myocardial contractive abnormalities before any clinical presentations [19]. In our study, Sa appeared to be a more-sensitive predictor of mortality than was the ejection fraction. However, the value of Sa remains to be validated in more patient populations [7,19-21], especially in critically ill patients. Moreover, unlike patients with cardiovascular diseases in whom a lower Sa was associated with lower survival rate, survivors with septic shock exhibited significantly decreased Sa, as seen in our study.

Many hypotheses have been proposed for myocardial depression in septic shock [2,22]. However, most of them could not explain why survivors exhibited more-marked myocardial depression. Levy *et al. *[23] demonstrated myocardial hibernation in sepsis by using magnetic resonance imaging, positron emission tomography, and single-photon emission computed tomography imaging. Myocardial hibernation is the best mechanism to preserve cardiac myocytes by downregulation of oxygen consumption and energy requirements. It is an adaptive response to maintain myocardial viability for prevention of cell-death pathway activation and to aid the future full recovery. The slightly increased cardiac biomarkers (that is, cTnI) in the study population also support that physical myocardial injury is negligible. Instead, the heart was injured "functionally." Such a potential beneficial response must be based on an assumption that tissue perfusion might be maintained with the depressed heart. With a close look at our data, lactate, a good marker of tissue perfusion [24], was not elevated, despite myocardial depression. However, serial echocardiographs were performed for only some of our patients; further serial study was warranted to support this hypothesis.

Persistent vasoplegia might be another explanation for our finding. Although the Sa has advantages over previously used measures of LV systolic function, such as LVEF, it still is load dependent, afterload especially [25,26]. In the study of Robotham *et al. *[26], the same level of LVEF may correspond to very different level of intrinsic LV contractility. For instance, an LVEF of 55% may correspond to severe impressed intrinsic LV contractility in the presence of decreased vascular tone. It would not be surprising to find relatively normal or supernormal Sa in nonsurvivors in our study, which reflected a hyperkinetic state associated with persistent and profound vasoplegia that, in turn, could be a marker of sustained cytokine release. This kind of persistent vasoplegia was associated with a high mortality rate, which is consistent with our findings.

Sturgess *et al. *[27] also reported the role of TDI to assess LV function in septic shock patients. They failed to find any difference in LV systolic function between survivors and nonsurvivors. This might be explained by the small sample size (*n *= 21) and high prevalence of cardiac diseases (43%) in the study population. Myocardial infarction may influence systolic and diastolic TDI values, as previously described by Alam *et al. *[28]. We also included patients with coronary heart disease in our study, but patients with myocardial infarction were excluded. Furthermore, after the exclusion of patients with coronary heart disease, the predictive value of Sa still remained. Similar to the study of Sturgess [27], Landesberg *et al. *[29] did not find evidence of LV systolic dysfunction in survivors. However, only 62% in the study population had septic shock. This, in addition to the imbalanced distribution of septic shock between survivors and nonsurvivors (57% versus 72%; *P *= 0.012), precluded direct comparison between their study result and that of our study.

In our cohort of patients, MAP was maintained at a higher level than that recommended by the guideline [30]. This can be explained by the high prevalence of hypertension in the study population. Although no evidence suggested the benefit of hyperdynamic support, Dünser *et al. *[31] reported that the time spent below the MAP of 75 mm Hg would increase the risk of subsequent renal-replacement therapy. Such findings suggest the importance of addressing ischemic acute renal failure in the absence of frank hypotension [32]. Accordingly, we try to individualize the target of blood pressure in our patients, based on usual level. The study of Vieillard-Baron *et al. *[17] suggested that the increased norepinephrine loads necessary to maintain high blood pressures were likely to cause LV hypokinesia. However, the dose of norepinephrine to maintain blood pressure in our groups was lower in survivors (0.3 (0.2 to 0.5) versus 0.6 (0.2 to 1.0) μg/kg/min], who had a higher incidence of LV hypokinesia. In the final multivariate analysis, even after adjustment for norepinephrine treatment, the prognostic value of Sa still attained statistical significance.

### Limitations

First, the sample size in our study was relatively small, but the robust association between Sa and mortality rate suggests that this was not just an accidental finding. Possible selection biases might exist because more patients were excluded than were studied. However, the baseline characteristics, except for coronary heart disease, did not show significant differences between the patients excluded and included. Second, this study is a single-center study. Our local management strategy may influence the patient's outcome, which might preclude the generalization of the study findings. Third, the potential confounding factors for TDI variables were not explored in this study. A more-detailed study focusing on these confounding factors is highly desirable. Fourth, correlation between TDI variables and blood flow-derived parameters was not performed because pulmonary artery catheters were inserted in only half of the study population.

## Conclusion

Our study demonstrated that LV systolic function, as determined by TDI, in particular, by Sa, might be associated with mortality in patients with septic shock. Concerning the limitations as discussed earlier, further studies are warranted to confirm our findings.

## Key messages

• In patients with septic shock, compared with nonsurvivors, survivors exhibited more marked myocardial depression.

• Evaluation of LV function by TDI, in particular, by Sa, might be associated with mortality in patients with septic shock.

## Abbreviations

APACHE: Acute Physiology And Chronic Health Evaluation; E: peak velocity of early diastolic transmitral flow; Ea: early diastolic velocity measured at the mitral annulus; EF: ejection fraction; ICU: intensive care unit; LV: left ventricular; LVEDV: LV end-diastolic volume; LVESV: LV end-systolic volume; PCWP: pulmonary capillary wedge pressure; ROC: receiver-operating characteristic; SIRS: systemic inflammatory response syndrome; SOFA: Sequential Organ Failure Assessment; TDI: tissue Doppler imaging; Sa: peak systolic velocity measured at the mitral annulus.

## Competing interests

The authors declare that they have no competing interests. All authors report no funding for support of this work.

## Authors' contributions

YL, LW, BD, JZ, XG, JP, XH, SZ, QF, and WZ participated in the design of the study and performed the statistical analysis. YL, LW, and BD conceived of the study and participated in its design and coordination. All authors read and approved the final manuscript.

## Supplementary Material

Additional file 1 **Supplement**. Supplement to Methods Results. Click here for file
