# Supplementary material for: The prognostic value of left ventricular systolic function measured by tissue Doppler imaging in septic shock
Source: Crit Care. 2012 May 3;16(3):R71. doi: 10.1186/cc11328 (PMC3580613; doi:10.1186/cc11328)
Supplement: Additional file 1 — Supplement. Supplement to Methods Results. [file cc11328-S1.DOC]

Additional file 1

Methods

Definitions:

1. SIRS criteria:
   1. Temperature > 38°C or < 36°C;
   2. Heart rate > 90 beats/min;
   3. White blood cell count > 12,000 or < 4000 cells/mm^3^;
   4. Respiratory rate > 20 breaths/min or PaCO_2_ < 32 mmHg
2. Severe sepsis: Sepsis plus evidence of organ dysfunction (i.e. oliguria, lactic acidosis, perfusion abnormalities, or alteration of mental status).
3. Septic shock: Severe sepsis plus one of the followings:
   1. Persistent hypotension, mean arterial pressure (MAP) < 60 mmHg (< 80 mmHg if history of hypertension) after 20–30 mL/kg starch or 40–60 mL/kg normal saline, or pulmonary capillary wedge pressure (PCWP) between 12 and 20 mmHg;
   2. Requirement of vasopressors after adequate fluid resuscitation.

Management strategy:

Our local management strategy in ICU as well as the emergency department conforms to international consensus guideline about sepsis management , except for target of mean arterial pressure . The mean arterial pressure of patient was maintained at usual level.

1. Resuscitation goals within 6 hours:
   1. Mean arterial pressure maintained at usual level
   2. Urine output ≥ 0.5 mL/kg/hr
   3. Mixed venous oxygen saturation≥ 65% or central venous oxygen saturation ≥ 70%
2. If resuscitation goal not achieved:
   1. Increase fluid infusion rate, if response to fluid challenge (fluid challenge 15-20 mL/kg crystalloids or colloids at equivalent dose) or signs of hypovolaemia at echocardiography
   2. Increase dose of vasopressor, if no response to fluid challenge or no signs of hypovolaemia at echocardiography
   3. Consider transfusion to maintain Hct > 30%, if mixed venous oxygen saturation ⩽ 65% or central venous oxygen saturation ⩽ 70% despite adequate preload and perfusion pressure
   4. Start inotropes, if if mixed venous oxygen saturation ⩽ 65% or central venous oxygen saturation ⩽ 70% despite adequate preload and perfusion pressure and Hct > 30%
   5. Consider pulmonary artery catheter monitoring whenever difficulty in judgment
      1. Fluid resuscitation to maintain pulmonary capillary wedge pressure 12 to 15 mmHg
      2. Consider vasopressor to maintain blood pressure at usual level if no responsive to fluid resuscitation
      3. Start inotropes, i.e. dobutamine or epinephrine if cardiac index < 2.5 L/min/m^2^, despite adequate preload and perfusion pressure

Results

Comparison between patients included and excluded：

Considering the clinical outcome should be confounded by the decision of withhold or withdraw, patients with decision of withdraw or withhold life-sustaining therapy were not included in this comparison. Excluded population showed significant higher proportion of coronary heart disease. (Supplement table 1)

Information about included patients with coronary heart disease (CAD)

Eight patients in our study had documented CAD of which the diagnosis was made by the cardiologists. All the eight patients had history of stress-related chest pain, coronary angiography was done in 5 patients and stenosis of more than 70% was found in at least 1 coronary artery (LAD, RCA or LCX) in these 5 patient; positive result of exercise-stress ECG was found in other 2 patients; and the changes of the ST-T segment in ECG was documented in 1 patient when the chest pain developed. All the eight patients underwent medical therapy for secondary prevention of CAD, all the patients were stable in the CAD and no ACS was diagnosed in the last 3 months prior to inclusion.

References:

1. Dellinger RP, Levy MM, Carlet JM, Bion J, Parker MM, Jaeschke R, Reinhart K, Angus DC, Brun-Buisson C, Beale R *et al*: **Surviving Sepsis Campaign: international guidelines for management of severe sepsis and septic shock: 2008**. *Intensive Care Med* 2008, **34**:17-60.

2. Takala J: **Should we target blood pressure in sepsis?** *Crit Care Med* 2010, **38**:S613-619.

Table S1, Baseline Characteristics and comparison between patients excluded* and included.

|  | Included  (n=61) | Excluded*  (n=57) | P value |
| --- | --- | --- | --- |
| Characteristics |  |  |  |
| Hospital death, n(%) | 24(39) | 26(46) | 0.491 |
| Age, yr | 65±15 | 65±19 | 0.926 |
| Male, n(%) | 33(54) | 33(58) | 0.678 |
| BMI, kg/m^2^ | 23±3 | 22±4 | 0.220 |
| APACHE IV score | 84±22 | 83±28 | 0.888 |
| APACHE IV predicted mortality, % | 40±24 | 42±29 | 0.770 |
| Comorbidities |  |  |  |
| Coronary Heart Disease, n(%) | 8(13) | 20(35) | 0.005 |
| Hypertension, n(%) | 28(46) | 23(40) | 0.543 |
| Diabetes, n(%) | 17(28) | 11(19) | 0.274 |
| Primary Diagnosis of Infection |  |  |  |
| Pneumonia, n (%) | 34(56) | 30(53) | 0.735 |
| Bacteremia, n (%) | 7(12) | 11(19) | 0.238 |
| Peritonitis, n (%) | 6(10) | 8(14) | 0.481 |
| Others, n (%) | 14(23) | 8(14) | 0.214 |

BMI=body mass index; APACHE=acute physiology and chronic health evaluation

* Patients with decision of withdraw or withhold were not in this group


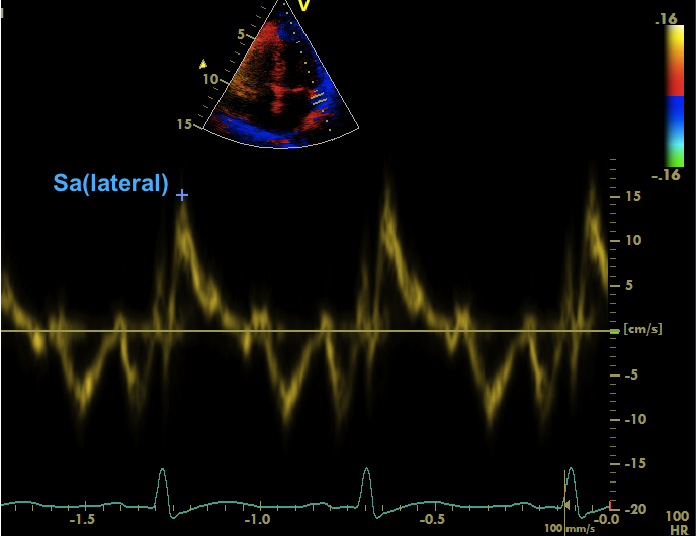


Figure S2: Pulse Doppler sample volume was placed at the septal and lateral MV annulus to get the average value of systolic (Sa) and early diastolic velocity (Ea).
